# Incidence of skin and soft tissue infections in general practice and out-of-hours services in Norway 2006–2022

**DOI:** 10.1080/02813432.2026.2649331

**Published:** 2026-03-27

**Authors:** Magnus Fossum, Ingrid Keilegavlen Rebnord, Knut Eirik Ringheim Eliassen, Leo Larsen, Guri Rørtveit, Knut-Arne Wensaas, Knut Erik Emberland

**Affiliations:** ^a^ Department of Global Public Health and Primary Care, University of Bergen, Bergen, Norway; ^b^ Director General’s Office, Norwegian Institute of Public Health, Oslo, Norway; ^c^ Research Unit for General Practice in Bergen, NORCE Norwegian Research Centre, Bergen, Norway

**Keywords:** Epidemiology, bacterial infections, impetigo, cellulitis, erysipelas, primary care

## Abstract

**Background:**

Most patients with skin and soft tissue infections (SSTIs) are treated in the primary care setting. However, recent epidemiologic data in primary care are limited.

**Objective:**

To investigate incidence and patient characteristics of SSTI episodes in daytime general practice (DGP) and out-of-hours (OOH) services in Norway.

**Methods:**

Registry-based study using reimbursement claims data from Norwegian primary care for the period 2006–2022. SSTI consultations were grouped into episodes, and patient morbidities were identified using a morbidity index. Incidence rates were calculated based on population data from Statistics Norway.

**Results:**

During the study period, 1 171 018 unique patients experienced altogether 1 727 736 SSTI episodes in Norwegian primary care. Most SSTI episodes (76.1%) were managed in DGP and incidence rates peaked in the third quarter. The overall annual incidence was 20.0/1000 inhabitants, with a decrease from 23.7/1000 inhabitants in 2006 to 17.0 in 2022. During the period, the largest decline among the SSTIs was observed for impetigo (68.1% decrease), infected wound or bite (46.0% decrease), and cellulitis and erysipelas (23.6% decrease), while abscess and furuncle and paronychia all remained stable. Overall, elderly patients and patients with a morbidity score of one or more had higher incidence rates of SSTIs, that increased over the period.

**Conclusion:**

The incidence of SSTIs in DGP and OOH services in Norway declined over the study period, except for elderly and persons with multimorbidity. As the population is increasingly older and multimorbid, such infections may represent a growing challenge in the future.

## Introduction

Skin and soft tissue infections (SSTIs) range from mild to life-threatening and are caused by bacteria invading normal skin or a compromised skin barrier due to cuts, bites, burns, needles or pre-existing skin conditions [1,2]. SSTIs are most frequently caused by *Streptococcus* and *Staphylococcus* species [3], and include the diagnoses cellulitis, erysipelas, impetigo, and skin abscesses such as furuncles and carbuncles [4–6]. Although common all year round, SSTI incidence often peaks during periods of higher temperatures and humidity, typically in late summer [5,7–9]. SSTIs are common in the healthy population, but are more frequent in children under five years of age and adults over 65 years, men, and obese individuals [10–12]. The presence of multiple chronic health conditions is also a known risk factor [10,13].

The global burden of bacterial skin diseases is substantial. According to the Global Burden of Disease data, incidence globally is estimated to have increased from 89.9/1000 inhabitants in 1990 to 108.2/1000 inhabitants in 2021 [14]. Although the incidence rate is highest in low socio-demographic index (SDI) regions, the disability-adjusted life years (DALYs) due to SSTIs has increased the most in high SDI regions and in the population aged >85 years. Studies of SSTI incidence in western countries have observed increasing incidence rates in the first decade of the twenty first century, whereas some recent studies indicate a decreasing trend [4,15–18]. However, most of these studies are from North America, or based on hospitalised patients or smaller subgroups of the population [19–21]. Data from primary care, where most patients are managed, is scarce. Updated incidence numbers for SSTIs, particularly from primary care, will improve our understanding of how these infections are distributed across patient characteristics and trends over time. Therefore, this study aims to present updated data on the incidence of SSTI episodes managed in daytime general practice (DGP) and out-of-hours (OOH) services in Norway from 2006 to 2022 and associated patient characteristics.

## Materials and methods

### Study design

We conducted an observational registry-based study using reimbursement claims data from 2006 to 2022. We included data from all doctor-patient encounters in DGP and OOH services with SSTI diagnoses according to the international classification of primary care, 2^nd^ edition (ICPC-2) [22].

### Setting and patients

All Norwegian residents are entitled to a general practitioner in the national primary health care service, with over 99% of the population registered with a general practitioner as of 2024^23^. Outside regular opening hours the municipalities organise OOH services that are usually staffed by on-call general practitioners. Doctors in DGP and OOH services send reimbursement claims electronically to the Norwegian Health Economics Administration (Helfo), including ICPC-2 diagnosis. Consultation data is automatically transferred to the Norwegian Control and Payment of Health Reimbursement Database (KUHR), part of the Norwegian Registry for Primary Health Care. Data from all encounters in the public DGP and OOH service are registered in KUHR.

### Data and variables

Data from all doctor-patient encounters in DGP and OOH services reimbursed from Helfo from 1 January 2006 to 31 December 2022 were available in the dataset. The variables included were ICPC-2 codes for diagnoses, patient sex and age, date of encounter and type of practice (DGP or OOH services). The time variable was quarter and year, where quarter was defined as January-March, April-June, July-September and October-December. We categorised age into ten categories: 0–4, 5–14, 15–24, 25–34, 35–44, 45–54, 55–64, 65–74, 75–84, and ≥85 years of age.

We defined in-person consultations, virtual consultations, and home visits as ‘consultations’, and communication by phone or through the reception as ‘simple contacts’. The start of an SSTI episode was defined as the first consultation with an SSTI diagnosis, and an encounter (consultation or simple contact) with any of the SSTI diagnoses occurring ≤30 days after the previous SSTI encounter was considered part of the same episode. A simple contact with any SSTI diagnosis after a consultation could extend the duration of an episode but was not considered an episode when occurring alone. If a patient was seen in both DGP and OOH services during an episode, the type of practice of the first consultation was used to define type of practice for the episode.

Relevant ICPC-2 codes were selected with reference to previous epidemiological studies on SSTIs that utilised similar data sources [4–6,9]. The codes included in this study were paronychia (S09), abscess and furuncle (S10), infected wound or bite (S11), cellulitis and erysipelas (S76), and impetigo (S84) (Table 1). Some SSTIs of interest cannot be identified by ICPC-codes, for instance surgical site infections and chronic ulcer infections, since the appropriate diagnoses (complication of medical treatment (A87) and chronic ulcer skin (S97)) will include non-infectious conditions as well. If a consultation or episode contained multiple SSTI diagnosis codes, the more severe infection was registered, based on clinical experience within the research group. The following diagnosis code hierarchy, from most to least severe, was used: (1) cellulitis and erysipelas; (2) abscess and furuncle; (3) infected wound or bite; (4) paronychia; and (5) impetigo.

**Table 1.** Skin and soft tissue infections (SSTIs) and morbidities* by international classification of primary care, 2^nd^ edition (ICPC-2) diagnosis codes.

Description	ICPC-2 codes
SSTIs	
Paronychia	S09
Abscess and furuncle	S10
Infected wound or bite	S11
Cellulitis and erysipelas	S76
Impetigo	S84
Morbidities*	
Cancer	A79, B72-74, D74-77, L71, N74, R84-85, T71, U75-77, W72, X75-77, Y77-78
Viral hepatitis	D72
Liver disease	D97
Heart failure	K77
Stroke and TIA	K89-90
Atherosclerosis and PVD	K92
Multiple sclerosis	N86
Parkinsonism	N87
Epilepsy	N88
Chronic alcohol abuse	P15
Substance abuse	P18-19
Learning disability	P24-25
Dementia	P70
Schizophrenia and psychosis	P72-73, P98
Anorexia and bulimia	P86
COPD	R95
Diabetes	T89-90
Glomerulonephritis	U88

TIA: transient ischemic attack; PVD: peripheral vascular disease; COPD: chronic obstructive pulmonary disease.

*Morbidity index by Sandvik et al.^23^.

Morbidity scores were identified using diagnosis codes from the ICPC morbidity index by Sandvik et al. validated for primary care registry data [23]. Consultations from 2006 to 2022 with any of 18 morbidities in the ICPC morbidity index were included (Table 1). A patient was given one point for each unique morbidity in at least one consultation in the year (365 days) prior. To ensure accuracy of the morbidity score, SSTI episodes in 2006 were excluded from the morbidity index incidence rate numbers to provide a run-in period. Following the ICPC morbidity index, patients were categorised as having a morbidity score of 0, 1, 2 or 3 or more at the time of each SSTI episode.

### Statistics

The data were analysed in the software Stata/SE 18.5, and Microsoft Excel 365 was used to create tables and graphs. The total number of patients was identified using unique pseudonymous personal identification numbers in the data. Incidence rates per 1000 inhabitants were calculated using population as an estimate of person years at risk. Population data was retrieved from Statistics Norway for 1 January each calendar year [24]. Ninety-five percent confidence intervals (CI) were calculated assuming a Poisson distribution, and trends over time were described based on visual examination of tables and graphs.

## Results

### SSTI patients

During the study period (2006–2022), there were 843 851 (72.1%) patients with one SSTI episode, 211 187 (18.0%) patients with two SSTI episodes and 115 980 (9.9%) patients with three or more SSTI episodes. In total there were 1 171 018 patients who experienced one or more SSTI episodes in DGP or OOH services from 2006 to 2022 (Table 2). The sex distribution was nearly equal for all the years studied, with females comprising 49.0% and males 51.0% of those with an SSTI. Most patients (86.6%) experiencing their first SSTI episode had a morbidity score of zero, while 11.2% had a score of one, 1.9% had a score of two, and only 0.3% had a score of three or more. Younger patients accounted for a larger proportion of patients with their first episode of SSTI, with those aged 5–14 years representing 12.7% and those aged 0–4 years representing 12.3%. The oldest age groups accounted for the smallest proportion of patients, with 8.9% of patients in the 65–74 age group, 6.4% in the 75–84 age group, and 3.2% aged 85 years or older. There were 22 576 consultations with multiple SSTI diagnoses, representing 0.9% of all consultations.

**Table 2.** Demographics of patients with first episode of skin and soft tissue infections (SSTIs) in Norwegian primary care, 2006–2022.

Category	No. of Patients With SSTIs	%
Total	1 171 018	
Sex		
Female	573 965	49.0
Male	597 053	51.0
Morbidity score		
0	927 435	86.6
1	119 792	11.2
2	20 417	1.9
≥3	3 266	0.3
Age group		
0–4	144 253	12.3
5–14	148 404	12.7
15–24	142 983	12.2
25–34	124 225	10.6
35–44	134 957	11.5
45–54	133 403	11.4
55–64	126 866	10.8
65–74	104 189	8.9
75–84	74 807	6.4
≥85	36 931	3.2

Morbidity score = number of patient morbidities last 365 days (2007–2022).

### SSTI incidence

**Figure 1.**
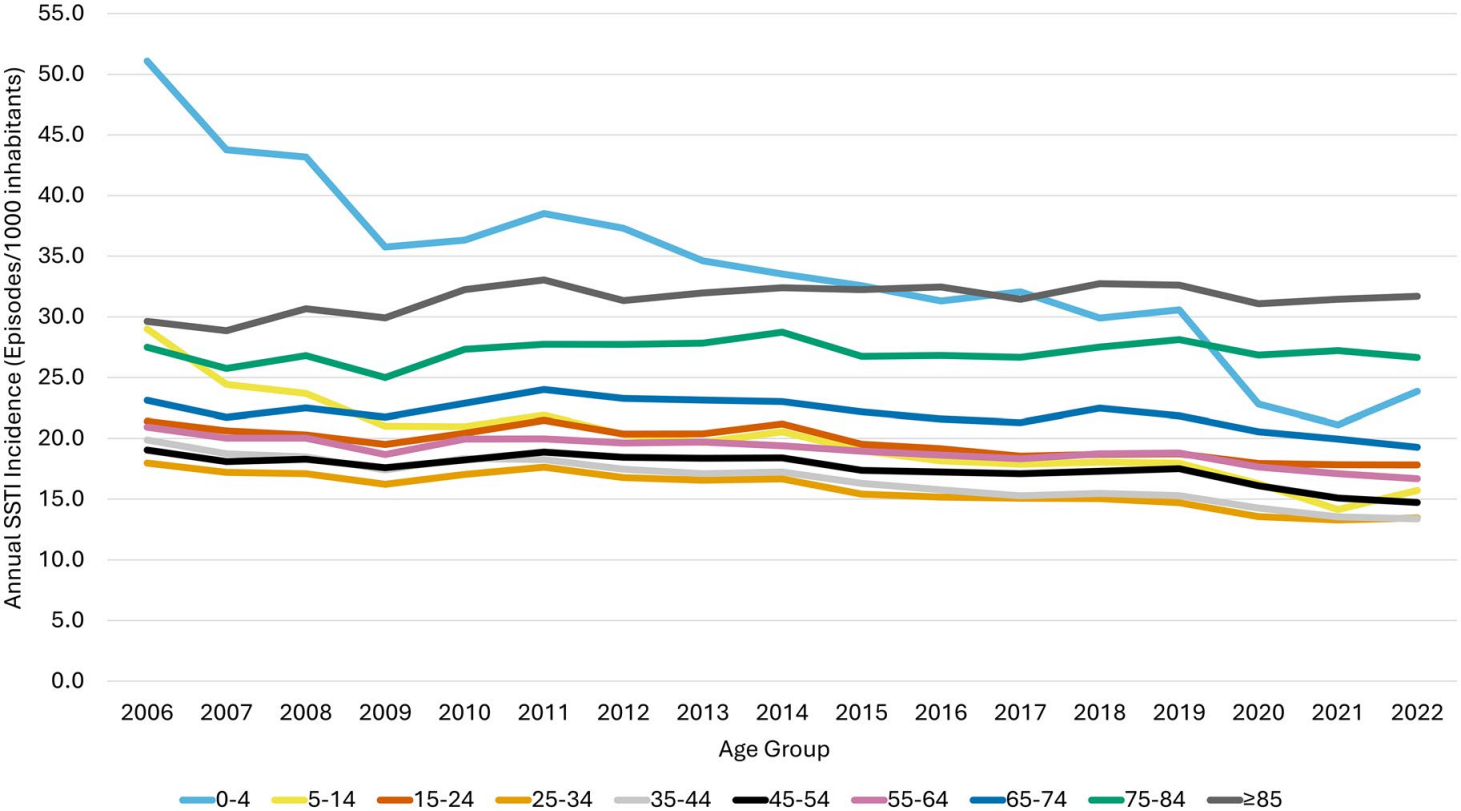
Annual incidence of skin and soft tissue infections (SSTIs) managed in Norwegian primary care by age group, 2006–2022.

During the study period there were a total of 3 143 905 encounters in DGP or OOH services with an SSTI diagnosis, consisting of 2 293 034 in-person consultations, 37 306 virtual consultations, 69 911 home visits, and 743 654 simple contacts. These encounters were aggregated into 1 727 736 SSTI episodes (Table 3), with an overall annual incidence rate of 20.0/1000 inhabitants (CI: 20.0–20.1). The incidence rate of SSTIs declined by 28.3% from 23.7/1000 inhabitants (CI: 23.7–24.0) in 2006 to 17.1/1000 inhabitants (CI: 17.0–17.2) in 2022 (Figure 1). The incidence rates were slightly higher among males (20.5/1000 inhabitants, CI: 20.4–20.5) compared to females (19.6/1000 inhabitants, CI: 19.6–19.6). Patients with a morbidity score of zero had an incidence rate of 18.7/1000 inhabitants (CI: 18.7–18.8), compared to 27.3 (CI: 27.1–27.4) among those with a score of one, 32.5 (CI: 32.1–32.8) for those with a score of two and 35.6 (CI: 34.8–36.4) for those with a score of three or more.

**Table 3.** Incidence of skin and soft tissue infections (SSTIs) managed in Norwegian primary care by patient characteristics, 2006–2022.

Characteristic	Episodes (*N*, %)		Person-years	Annual Incidence Rate of SSTIs/1000 Inhabitants (95% CI)
Total	1 727 736	(100)	86 228 635	20.0 (20.0–20.1)
Sex				
Female	841 814	(48.7)	42 966 647	19.6 (19.6–19.6)
Male	885 922	(51.3)	43 261 988	20.5 (20.4–20.5)
Morbidity score				
0	1 353 284	(83.7)	72 217 199	18.7 (18.7–18.8)
1	214 468	(13.3)	7 870 067	27.3 (27.1–27.4)
2	41 847	(2.6)	1 289 413	32.5 (32.1–32.8)
≥3	7 539	(0.5)	211 737	35.6 (34.8–36.4)
Age group				
0–4	173 147	(10.0)	5 083 070	34.1 (33.9–34.2)
5–14	211 108	(12.2)	10 616 158	19.9 (19.8–20.0)
15–24	214 599	(12.4)	10 936 514	19.6 (19.5–19.7)
25–34	182 167	(10.5)	11 584 636	15.7 (15.7–15.8)
35–44	201 236	(11.7)	12 112 672	16.6 (16.5–16.7)
45–54	205 839	(11.9)	11 783 620	17.5 (17.4–17.5)
55–64	194 110	(11.2)	10 233 155	19.0 (18.9–19.1)
65–74	165 442	(9.6)	7 549 931	21.9 (21.8–22.0)
75–84	119 647	(6.9)	4 413 692	27.1 (27.0–27.3)
≥85	60 441	(3.5)	1 915 187	31.6 (31.3–31.8)
Quarter				
January-March	392 772	(22.7)	21 557 159	18.2 (18.2–18.3)
April-June	421 189	(24.4)	21 605 314	19.5 (19.4–19.6)
July-September	492 043	(28.5)	21 657 011	22.7 (22.7–22.8)
October-December	421 732	(24.4)	21 724 711	19.4 (19.4–19.5)
Type of practice				
OOH services	412 970	(23.9)		
DGP	1 314 766	(76.1)		

CI: confidence interval; OOH: out-of-hours services; DGP: daytime general practice.

The highest age-specific incidence rate was observed in children aged 0–4 years (34.1/1000 inhabitants, CI: 33.9–34.2) and those aged ≥85 years (31.6/1000 inhabitants, CI: 31.3–31.8). All age groups between 0 and 74 years exhibited some degree of decline in SSTI incidence, although the largest decline was observed among those 0–4 years (53.2% decrease) and 5–14 years old (45.9% decrease). In contrast, the annual SSTI incidence among the ≥85-year-olds increased 7.1% from 29.6 to 31.7 per 1000 inhabitants from 2006 to 2022 and the incidence among the 75–84-year-olds remained relatively stable (Figure 1).

Seasonal variation was observed, with the highest quarterly incidence of episodes during the third quarter July-September (22.7/1000 inhabitants, CI: 22.7–22.8), compared to the lowest rates during the winter months January-March (18.2/1000 inhabitants, CI: 18.2–18.3). The proportion of episodes in each type of practice remained relatively stable during the period, with the majority of SSTI episodes ­managed in DGP (76.1%), while OOH services accounted for 23.9% of episodes (Figure 1).

### SSTI diagnoses

**Figure 2.**
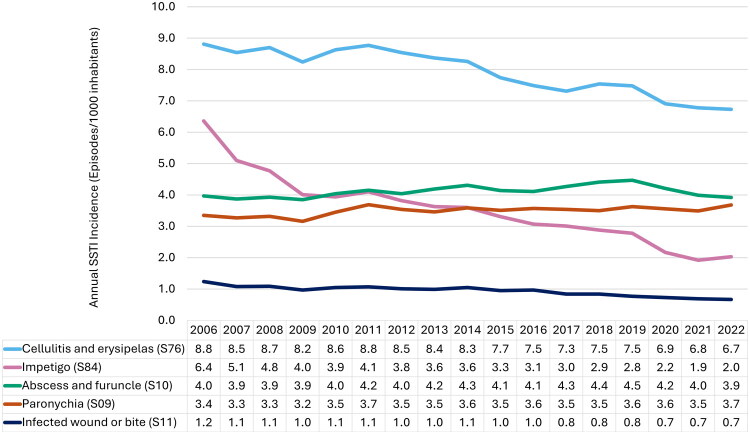
Annual incidence of skin and soft tissue infections (SSTIs) managed in Norwegian primary care, 2006–2022.

Throughout the study period, cellulitis and erysipelas (S76) consistently represented the highest proportion of SSTIs (Figure 2). Abscess and furuncle (S10) and paronychia (S09) were the second and third most common SSTIs for most of the period, followed by impetigo (S84). The lowest incidence rates were observed for infected wound or bite (S11).

Incidence of cellulitis and erysipelas declined with 23.6%, from 8.8/1000 inhabitants (CI: 8.7–8.9) in 2006 to 6.7/1000 inhabitants (CI: 6.7–6.8) in 2022. Impetigo showed the largest decline over the study period, dropping 68.1% from 6.4/1000 inhabitants (CI: 6.3–6.4) to 2.0/1000 inhabitants (CI: 2.0–2.1). Meanwhile, abscess and furuncle incidence remained relatively stable, fluctuating between 3.9 and 4.5/1000 inhabitants, while paronychia ranged from 3.2 to 3.7/1000 inhabitants. The incidence of infected wound or bite dropped from 1.2 to 0.7/1000 inhabitants, a 46.0% decline over the study period.

## Discussion

### Key results

There were altogether 1 727 736 episodes for SSTIs in Norwegian primary care over the 17-year period from 2006 to 2022, with an overall annual incidence rate of 20.0/1000 inhabitants. Most SSTI episodes (76.1%) were managed in DGP and incidence rates peaked in the third quarter. We observed a substantial decline in SSTI incidence (28.3% decrease) during the study period, with notable reductions observed for impetigo (68.1% decrease), infected wound or bite (46.0% decrease) and cellulitis and erysipelas (23.6% decrease), while abscess and furuncle and paronychia incidence remained relatively stable. Incidence rates were slightly higher among males and there was a clear association between morbidity score and SSTI incidence. The highest incidence rates of SSTIs were seen in the youngest (0–4 years) and oldest (≥85 years) patient groups. All age groups below 75 years showed decreasing SSTI incidence rates during the period, with the most substantial declines in children 0–4 years (53.2% decrease) and 5–14 years (45.9% decrease).

### Strengths and limitations

The reimbursement claims registry provides a long follow-up period and a nearly complete data set of all doctor-patient encounters in DGP and OOH services in Norway. This provides a unique opportunity for epidemiological studies. As the KUHR registry only covers reimbursement claims from GPs and OOHs, patients with SSTIs treated in primary care institutions like nursing homes are not included in our data. However, this a small group that in 2022 accounted for 10.6% of those over 80 years of age [25].

The ICPC-2 code system also presents limitations, as it is not always detailed enough to differentiate between SSTI diagnoses. Potentially relevant overlapping codes could be insect bite/sting (S12) or animal/human bite (S13). However, these codes should be coded as infected wound or bite (S11) when infection is present and thereby included in our data. To our knowledge there are no studies on the distribution of diagnoses within each diagnosis code in the ICPC-2 coding system. The possibility for misclassification and uncertainty of diagnosis codes also applies to the morbidity index used in this study. Codes for viral or fungal infections were excluded, as the focus of this study was on bacterial skin infections.

We have chosen to only include SSTI episodes resulting in a consultation, excluding episodes with only simple contacts. This could potentially result in underreporting the total number of episodes, but reduces the risk of misclassification that we consider to be substantial as it is unlikely that many SSTI episodes resulting in medical care are managed exclusively through simple contacts alone.

### Interpretation

We found an overall SSTI incidence rate of 20.0/1000 inhabitants in DGP and OOH services in Norway from 2006 to 2022. The incidence observed in this study is relatively low compared to findings in other countries, although direct comparisons are limited by differences in healthcare systems, data sources, and case definitions.

Few recent studies have examined SSTI incidence over long periods, particularly in the European primary care setting. In North America, multiple studies based on insurance claims data have reported higher annual incidence rates: 24.0/1000 inhabitants (2008 to 2013) [18], 32.1–48.1 (1997–2005) [26], 40.1 (2001 to 2009) [15], 48.0 (2005 to 2010) [4], 49.6 (2009 to 2011) [17], 68.7 (1996 to 2008) [16] and 77.5 (2010 to 2020) [6]. A study from a health clinic in Fiji found an even higher annual incidence rate of 108.3/1000 inhabitants from 2018 to 2019, although this included scabies in addition to SSTIs [27]. Differences in patient risk factors, environmental conditions, healthcare-seeking behaviour, and the organisation of healthcare systems likely contribute to regional variation in SSTI incidence.

In Europe, a study using English and Welsh primary care data from 2006 reported an SSTI incidence of 33.5/1000 inhabitants [28], while Swedish primary care data from 2008 to 2013 found annual SSTI consultation rates from 49 to 64/1000 inhabitants [29]. A study of 45 primary care practices in the Netherlands (2007–2010) found that from 2007 to 2010 the incidence of cellulitis and erysipelas (S76) episodes increased from 8.1 in 2007 to 9.4/1000 inhabitants in 2010. The incidence of cellulitis and erysipelas in our study increased from 8.5 in 2007 to 8.6/1000 inhabitants in 2010, a similar incidence to the study from the Netherlands.

A possible contributor to the higher incidence observed in some of the studies from North America is the inclusion of postoperative infections, mastitis, and chronic ulcers in the SSTI diagnostic category. In our study, we wanted to focus on bacterial SSTIs, and for these conditions the ICPC-2 diagnosis codes encompass a mix of infectious and non-infectious conditions. However, the distribution of diagnoses in this study closely resembled the distribution of comparable diagnosis codes in previous studies using ICD-9 or ICD-10 [4,6,17]. It is also important to note that most of the studies we have compared with also include hospitalised patients [4,6,15,17]. Nevertheless, hospitalisation accounted for only a small proportion of SSTI episodes in those studies, ranging from 2.9% [6], 4.6% [4], to 15.8% [5], and is unlikely to explain the majority of the observed differences. The study reporting the highest SSTI incidence rate also included chronic ulcers, which made up 17.4% of all episodes [6], likely contributing significantly to the elevated rate. Therefore, it is likely that the difference may be at least partly due to higher incidence rates of similar diagnoses in North America compared to Norway.

Due to demographic shifts toward an aging population and increasing prevalences of chronic health conditions that are known risk factors, the incidence of SSTIs is expected to increase [1,10,13,30]. Studies in the early twenty first century observed an increasing trend in SSTIs [15,16,26], followed by a stable period after around 2007 [4,15,17]. Interestingly, a recent study on US insurance claims data (2010 to 2020) found a decreasing trend from 2010 to 2016 and a large decrease in 2020^6^. However, in the study most of the increase in SSTI incidence from 2016 to 2020 was caused by increasing incidence of chronic ulcers. Our study found that the incidence rate of SSTIs remained stable from 2007 to 2014, followed by a decline beginning in 2014 and a further decline during the COVID-19 pandemic years (2020–2022). The number of consultations per inhabitant in DGP and OOH services in Norway increased from the pre-pandemic years 2018 and 2019, driven by the surge in virtual consultations [31]. The observed decline in SSTI incidence during the pandemic did not follow the overall trend of increasing number of consultations per inhabitant. This may be attributed to reduced interpersonal contact, resulting in lower transmission rates, as well as changes in healthcare-seeking behaviour.

Although we observed a decrease in the overall incidence of SSTIs, an increasing proportion of the SSTI patient population is elderly. This trend may reflect the growing proportion of elderly patients living with chronic conditions predisposing these SSTIs [1,10,13,30]. The observed variations in SSTI incidences across sex, age, and morbidity score may also partially reflect differences in healthcare-seeking behaviour. Given the observational nature of our study, the analysis of patient characteristics may be influenced by unmeasured confounders.

In our study, we observed a substantial decrease in the incidence of impetigo from 2006 to 2022. This reflects the same downward trend in impetigo incidence seen since the impetigo epidemic in Norway from 2002 to 2004 [32–34] and shows that the incidence has continued to decline in recent years. It is important to note that our incidence figures represent only cases managed in DGP and OOH services; patients who do not seek health care services for medical treatment remain uncaptured in these statistics, potentially underestimating the true incidence rate in the community.

We observed a clear seasonal variation in SSTIs, with the highest incidence rates observed in the third quarter, corresponding with the trend observed in previous studies. The effect of the summer months is likely due to warmer temperatures and increased humidity inducing bacterial growth and transmission, and more outdoor activities and skin injuries that are conducive to infection [5,8,9]. The data showed a clear correlation between increasing morbidity score and incidence rates, similar to a recent study of SSTIs in the US [6].

## Conclusion

The incidence of SSTIs in Norwegian DGP and OOH services has decreased from 2006 to 2022. Incidence rates were highest among young children and the oldest adults. The only age group with an increase in incidence rate over the period was among the ≥85-year-olds. SSTI incidence increased with higher morbidity scores and showed a clear seasonal variation, with higher incidence rates in the third quarter. Cellulitis and erysipelas were the most common diagnoses, and along with impetigo and infected wound or bite, a substantial reduction in incidence was observed for all these infections. Abscess and furuncle, together with paronychia, represented a substantial proportion of SSTIs and a stable incidence was observed for these infections.

## Supplementary Material

Supplemental Material

## Data Availability

The data used in this article cannot be shared due to limitations given by the ethical approval granted by the Regional Ethics Committee.
