# Supplementary material for: Incidence of skin and soft tissue infections in general practice and out-of-hours services in Norway 2006–2022
Source: Scand J Prim Health Care. 2026 Mar 27;44(1):2649331. doi: 10.1080/02813432.2026.2649331 (PMC13034701; doi:10.1080/02813432.2026.2649331)
Supplement: Supplemental Material [file IPRI_A_2649331_SM2374.docx]

**Supplementary table S1** Annual incidence of skin and soft tissue infections (SSTIs) managed in Norwegian primary care by age group, 2006-2022

| **Annual Incidence of SSTIs (Episodes/1000 inhabitants)** | | | | | | | | | | | | | | | | | | |
| --- | --- | --- | --- | --- | --- | --- | --- | --- | --- | --- | --- | --- | --- | --- | --- | --- | --- | --- |
| **Age group** | **0-4** | 51.1 | 43.8 | 43.2 | 35.8 | 36.3 | 38.5 | 37.3 | 34.6 | 33.5 | 32.6 | 31.3 | 32.1 | 29.9 | 30.6 | 22.8 | 21.1 | 23.9 |
|  | **5-14** | 29.0 | 24.4 | 23.7 | 21.0 | 21.0 | 21.9 | 20.3 | 19.7 | 20.5 | 19.0 | 18.2 | 17.9 | 18.1 | 18.0 | 16.3 | 14.1 | 15.7 |
|  | **15-24** | 21.4 | 20.6 | 20.3 | 19.5 | 20.4 | 21.5 | 20.4 | 20.4 | 21.2 | 19.5 | 19.2 | 18.6 | 18.7 | 18.7 | 18.0 | 17.8 | 17.8 |
|  | **25-34** | 18.0 | 17.2 | 17.1 | 16.2 | 17.1 | 17.6 | 16.8 | 16.6 | 16.7 | 15.4 | 15.2 | 15.1 | 15.1 | 14.7 | 13.6 | 13.3 | 13.5 |
|  | **35-44** | 19.9 | 18.8 | 18.5 | 17.4 | 18.4 | 18.3 | 17.5 | 17.1 | 17.2 | 16.3 | 15.8 | 15.3 | 15.5 | 15.3 | 14.3 | 13.6 | 13.4 |
|  | **45-54** | 19.1 | 18.1 | 18.3 | 17.6 | 18.2 | 18.9 | 18.5 | 18.4 | 18.4 | 17.4 | 17.2 | 17.1 | 17.3 | 17.5 | 16.1 | 15.1 | 14.7 |
|  | **55-64** | 20.9 | 20.0 | 20.0 | 18.7 | 20.0 | 20.0 | 19.6 | 19.7 | 19.4 | 19.0 | 18.6 | 18.3 | 18.7 | 18.8 | 17.7 | 17.1 | 16.7 |
|  | **65-74** | 23.1 | 21.7 | 22.5 | 21.8 | 22.9 | 24.0 | 23.3 | 23.1 | 23.0 | 22.2 | 21.6 | 21.3 | 22.5 | 21.8 | 20.6 | 20.0 | 19.3 |
|  | **75-84** | 27.5 | 25.8 | 26.8 | 25.0 | 27.3 | 27.7 | 27.7 | 27.8 | 28.7 | 26.7 | 26.8 | 26.7 | 27.5 | 28.1 | 26.8 | 27.2 | 26.6 |
|  | **≥85** | 29.6 | 28.9 | 30.7 | 29.9 | 32.3 | 33.0 | 31.3 | 32.0 | 32.4 | 32.2 | 32.5 | 31.4 | 32.7 | 32.6 | 31.1 | 31.5 | 31.7 |
|  | **All age**  **groups** | 23.7 | 21.9 | 21.8 | 20.2 | 21.1 | 21.8 | 21.0 | 20.6 | 20.8 | 19.7 | 19.2 | 19.0 | 19.2 | 19.1 | 17.6 | 16.9 | 17.0 |
|  |  | **2006** | **2007** | **2008** | **2009** | **2010** | **2011** | **2012** | **2013** | **2014** | **2015** | **2016** | **2017** | **2018** | **2019** | **2020** | **2021** | **2022** |
|  |  |  |  |  |  |  |  |  | **Year** |  |  |  |  |  |  |  |  |  |
